# Diagnostic performance and optimal cut-off scores of the Massachusetts youth screening instrument-second version in a sample of Swiss youths in welfare and juvenile justice institutions

**DOI:** 10.1186/s12888-017-1197-2

**Published:** 2017-02-08

**Authors:** Claudia Dölitzsch, Laura E. W. Leenarts, Klaus Schmeck, Jorg M. Fegert, Thomas Grisso, Marc Schmid

**Affiliations:** 1 Klinik für Kinder- und Jugendpsychiatrie/Psychotherapie, Universitätsklinikum, Ulm, Germany; 2 Kinder- und Jugendpsychiatrische Klinik, Universitäre Psychiatrische Kliniken (UPK), Basel, Switzerland; 3 William Schrikker - Center of Research and Expertise, Amsterdam, The Netherlands; 4 Department of Psychiatry, University of Massachusetts Medical School, Worcester, USA

**Keywords:** Mental health screening, MAYSI-2, Psychiatric disorders, Juvenile justice, Gender

## Abstract

**Background:**

There is a growing consensus about the importance of mental health screening of youths in welfare and juvenile justice institutions. The Massachusetts Youth Screening Instrument-second version (MAYSI-2) was specifically designed, normed and validated to assist juvenile justice facilities in the United States of America (USA), in identifying youths with potential emotional or behavioral problems. However, it is not known if the USA norm-based cut-off scores can be used in Switzerland. Therefore, the primary purpose of the current study was to estimate the diagnostic performance and optimal cut-off scores of the MAYSI-2 in a sample of Swiss youths in welfare and juvenile justice institutions. As the sample was drawn from the French-, German- and Italian-speaking parts of Switzerland, the three languages were represented in the total sample of the current study and consequently we could estimate the diagnostic performance and the optimal cut-off scores of the MAYSI-2 for the language regions separately. The other main purpose of the current study was to identify potential gender differences in the diagnostic performance and optimal cut-off scores.

**Methods:**

Participants were 297 boys and 149 girls (mean age = 16.2, SD = 2.5) recruited from 64 youth welfare and juvenile justice institutions (drawn from the French-, German- and Italian-speaking parts of Switzerland). The MAYSI-2 was used to screen for mental health or behavioral problems that could require further evaluation. Psychiatric classification was based on the Schedule for Affective Disorders and Schizophrenia for School-Age Children, Present and Lifetime version (K-SADS-PL). The MAYSI-2 scores were submitted into Receiver-Operating Characteristic (ROC) analyses to estimate the diagnostic performance and optimal ‘caution’ cut-off scores of the MAYSI-2.

**Results:**

The ROC analyses revealed that nearly all *homotypic* mappings of MAYSI-2 scales onto (cluster of) psychiatric disorders revealed above chance level accuracy. The optimal ‘caution’ cut-off scores derived from the ROC curve for predicting (cluster of) psychiatric disorders were, for several MAYSI-2 scales, comparable to the USA norm-based ‘caution’ cut-off scores. For some MAYSI-2 scales, however, higher optimal ‘caution’ cut-off scores were found.

**Conclusions:**

With adjusted optimal ‘caution’ cut-off scores, the MAYSI-2 screens potential emotional or behavioral problems well in a sample of Swiss youths in welfare and juvenile justice institutions. However, as for choosing the optimal ‘caution’ cut off score for the MAYSI-2, both language as well as gender seems to be of importance. The results of this study point to a compelling need to test the diagnostic performance and optimal ‘caution’ cut-off scores of the MAYSI-2 more elaborately in larger differentiated language samples in Europe.

## Background

As the majority of youths residing in welfare as well as juvenile justice institutions have been shown to meet criteria for one or more psychiatric disorders [1–4], there is a growing consensus about the importance of mental health screenings of these youths [5]. For example, the prevalence of mental health disorders in a German youth welfare sample (*n* = 689) was 60%, with a predominance of disruptive and externalizing disorders [6]. A systematic review of 25 psychiatric surveys, including 16 750 detained adolescents, found that 11% of the boys and 29% of the girls had a depressive disorder. Conduct disorder was the most common of the studied disorders, slightly more than 50% and similarly prevalent across sexes [2]. In addition, according to a previous study [7] 74% of youths in welfare and juvenile justice institutions (*n* = 483) in Switzerland met criteria for psychiatric disorders (i.e., bipolar disorder, anxiety disorder, conduct disorder, major depression and substance abuse); in addition, 60% of these fulfilled criteria for more than one diagnosis. Evidently, similar types and rates of mental health problems are seen across welfare as well as juvenile justice populations.

Although adolescent delinquency is still a predominantly male phenomenon, an increasing number of girls are entering youth welfare and juvenile justice institutions [8, 9]. Available research suggests that girls entering youth welfare and juvenile justice institutions show higher prevalence rates of psychiatric disorders than boys (e.g., [5, 10]. In a Swiss sample [7] girls were significantly more likely to report affective, anxiety and trauma-related disorders, compared to boys. Whereas boys, were more likely to meet criteria for disruptive disorders (i.e., hyperkinetic, oppositional and conduct disorders). Consequently, it is recognized that the mental health problems of these youths need to be identified efficiently and reliably. With better information about the mental needs of these youths; welfare and juvenile justice institutions can identify, offer and evaluate treatment services intended to reduce the mental health problems and subsequently improve rehabilitative efforts of this vulnerable group [10–12].

Many instruments have been developed to screen for mental health problems in youths (e.g., [13–16]. However, these instruments may present difficulties when used in juvenile justice youths as, for example, they may be very time consuming, or do not assess for some of the most important problems for which screening in juvenile justice youths is needed (e.g., suicide risk, alcohol and substance use) or they assess only one disorder [17]. Based on the need for an appropriate screening tool, Grisso and Barnum [17] developed the Massachusetts Youth Screening Instrument-second version (MAYSI-2). The MAYSI-2 was specifically designed, normed and validated to assist juvenile justice facilities in the United States of America (USA), in identifying youths with potential emotional or behavioral problems such as suicidal and aggressive behavior that could require further specific and narrowly focused (psychiatric) evaluation [18, 19]. The instrument was released in 2000, and is now widely used in the juvenile justice system in about 44 states in the USA [18] and has shown to be reliable and valid in diverse samples of detained youths (e.g., [20–22]. The MAYSI-2 has also been extensively implemented into the juvenile justice system in Europe [19, 23, 24]. The MAYSI-2 is a self-report measure and contains seven scales (i.e., alcohol/drug use, angry-irritable, depressed-anxious, somatic complaints, suicide ideation, traumatic experiences and thought disturbance). The current cut-off scores (to identify youths with a *clinically relevant* score or to identify youths *most in need of attention*), which are also used in Europe, are based on the results of the *USA National Norms Study for the MAYSI-2* [17]. However, among the developed and Western nations, the USA has one of the most extreme and harsh juvenile justice systems in the world. The USA juvenile justice system is characterized by a higher level of violence, harsh punishment, and more incarceration for youths [25]. As a consequence, the settings and the target populations for which the MAYSI-2 was intended may differ between the USA and European countries, and therefore it is not known if these USA norm-based cut-off scores can be used in Switzerland.

For reasons mentioned above, the primary purpose of the current study was to estimate the diagnostic performance and optimal cut-off scores of the MAYSI-2 in a sample of Swiss youths in welfare and juvenile justice institutions. To our knowledge, the current study is the first study which addresses the optimal cut-off scores of the MAYSI-2 in a European sample. As the sample was drawn from the French-, German- and Italian-speaking parts of Switzerland, the three languages were represented in the total sample of the current study and consequently we could estimate the diagnostic performance and the optimal cut-off scores of the MAYSI-2 for the language regions separately. As gender may influence the accuracy of self-report measures (e.g., girls tend to reveal their feelings on self-report scales more readily than boys) it is reasonable to suggest that the current cut-off scores under- or over-detect certain disorders in boys or girls [17, 26]. Hence, the other main purpose of the current study was to identify potential gender differences in the diagnostic performance and optimal cut-off scores of the MAYSI-2 in a sample of Swiss youths in welfare and juvenile justice institutions.

## Methods

In the current study the same procedure, participants and assessment tools were used as in our earlier study [27].

## Statistics

Analyses were performed using Statistical Package for Social Sciences (SPSS) version 21. First, descriptive statistics for the subsamples were calculated. Second, the MAYSI-2 scores were submitted into Receiver-Operating Characteristic (ROC) analyses to estimate the diagnostic performance and optimal ‘caution’ cut-off scores of the MAYSI-2 [27]. Following a study by Wasserman et al. [22], we similarly related MAYSI-2 scales to (cluster of) psychiatric disorders [27]. We calculated sensitivity rates, which reflect the probability that when a psychiatric disorder is diagnosed, the MAYSI-2 will score as such. We calculated specificity rates, which reflect the probability that when a psychiatric disorder is *not* diagnosed, the MAYSI-2 will score as such. We also calculated positive (i.e., the probability that a psychiatric disorder will be diagnosed when the adolescent scores at or above the MAYSI-2 ‘caution’ cut-off score) and negative (i.e., the probability that a psychiatric disorder will *not* be diagnosed when the adolescent scores below the MAYSI-2 ‘caution’ cut-off score) predictive values. Furthermore, we calculated the area under the curve (AUC) - with higher AUC values reflect a higher probability that an adolescent with a disorder on the K-SADS-PL will score at or above the ‘caution’ cut-off on the designated MAYSI-2 scale. Significant AUC values of 1 reflect perfect accuracy, and significant AUC values greater than .50 reflect above chance-level accuracy [22]. Ideally, a screening instrument should have a significant AUC value of at least .70 or .90, indicating the instrument is adequately precise [28]. Finally, we calculated the optimal ‘caution’ cut-off scores. Because the MAYSI-2 cut-off scores are considered to be highest in both the sensitivity and specificity value [17], the optimal ‘caution’ cut-off scores are based on the difference between the sensitivity and specificity value to find the ‘caution’ cut-off score with the lowest difference between both values.

As we were interested in differences between the language regions and possible gender differences in the diagnostic performance and optimal ‘caution’ cut-off scores of the MAYSI-2, we performed analyses on five separate subsamples: total sample, German-speaking subsample, French/Italian-speaking subsample, boys and girls.

## Results

For detailed information about the descriptive statistics we refer to our earlier study [27]. The results of the ROC analyses on the total sample (Table 1) showed that the alcohol/drugs use scale accurately identified any substance use disorder. Any disruptive disorder was accurately identified by the angry-irritable scale. The depressed-anxious scale accurately identified any affective disorder. Any anxiety disorder was accurately identified by the depressed-anxious and the somatic complaints scales. The suicide ideation scale accurately identified suicide ideation/suicide attempts. In the total sample, compared to the current ‘caution’ cut-off scores, deviant optimal ‘caution’ cut-off scores were found for the depressed-anxious scale (optimal ‘caution’ cut-off score with regard to any affective: 4; optimal ‘caution’ cut-off score with regard to any anxiety: 4; current ‘caution’ cut-off score: 3).

**Table 1:** Receiver operating characteristics estimates and optimal ‘caution’ cut-off scores of MAYSI-2 scales (Total Sample)

Total sample					
	Sens/Spec	PPV/NPV	AUC (95% CI)	Optimal cut-off^a^	Current cut-off^b^
Any substance use (K-SADS-PL)					
MAYSI-2 alcohol/drug use	.76/.75	.35/.95	.83*** (.79–.87)	4	4
Any disruptive behavior (K-SADS-PL)					
MAYSI-2 angry-irritable	.59/.55	.54/.60	.61*** (.55–.66)	5	5
Any affective (K-SADS-PL)					
MAYSI-2 depressed-anxious	.52/.63	.21/.88	.62*** (.56–.69)	4	3
MAYSI-2 somatic complaints	.58/.48	.17/.86	.53 ns (.46–.60)	(2)	3
Any anxiety (K-SADS-PL)					
MAYSI-2 depressed-anxious	.63/.64	.17/.93	.67*** (.59–.74)	4	3
MAYSI-2 somatic complaints	.58/.68	.18/.93	.67*** (.60–.74)	3	3
Suicide ideation/suicide attempts (K-SADS-PL)					
MAYSI-2 suicide ideation	.74/.67	.39/.90	.75*** (.70–.81)	2	2

*MAYSI-2* Massachusetts Youth Screening Instrument-second version, *K-SADS-PL* Schedule for Affective Disorders and Schizophrenia for School-Age Children Present and Lifetime version, *Sens* sensitivity rate, *Spec* specificity rate, *PPV* positive predictive value, *NPV* negative predictive value, *AUC* area under the curve, *CI* confidence interval

*** *p* < .001; *ns* not significant

^a^The optimal ‘caution’ cut-off scores are based on the difference between the sensitivity and specificity value to find the cut-off score with the lowest difference between both values

^b^The current cut-off scores are based on the results of the USA National Norms Study for the MAYSI-2 [17]

ROC analyses on the German-speaking subsample (Table 2) revealed comparable results with the ROC analyses on the total sample. The alcohol/drugs use scale accurately identified any substance use disorder. Any disruptive disorder was accurately identified by the angry-irritable scale. The depressed-anxious scale accurately identified any affective disorder. Any anxiety disorder was accurately identified by the depressed-anxious and the somatic complaints scales. The suicide ideation scale accurately identified suicide ideation/suicide attempts. In the German-speaking subsample, compared to the current ‘caution’ cut-off scores, deviant optimal ‘caution’ cut-off scores were found for depressed-anxious (optimal ‘caution’ cut-off score with regard to any anxiety: 4; current ‘caution’ cut-off score: 3) and suicide ideation (optimal ‘caution’ cut-off score with regard to suicide ideation/suicide attempts: 3; current ‘caution’ cut-off score: 2).

**Table 2:** Receiver operating characteristics estimates and optimal ‘caution’ cut-off scores of MAYSI-2 scales (German-speaking and French/Italian-speaking Subsample)

	Sens/Spec	PPV/NPV	AUC (95% CI)	Optimal cut-off^a^	Current cut-off^b^
German-speaking subsample					
Any substance use (K-SADS-PL)					
MAYSI-2 alcohol/drug use	.77/.72	.34/.95	.83*** (.78–.87)	4	4
Any disruptive behavior (K-SADS-PL)					
MAYSI-2 angry-irritable	.58/.64	.67/.55	.65*** (.60–.71)	5	5
Any affective (K-SADS-PL)					
MAYSI-2 depressed-anxious	.64/.61	.19/.92	.61* (.51–.70)	3	3
MAYSI-2 somatic complaints	.48/.54	.13/.87	.50 ns (.41–.59)	(2)	3
Any anxiety (K-SADS-PL)					
MAYSI-2 depressed-anxious	.63/.70	.11/.97	.69** (.55–.82)	4	3
MAYSI-2 somatic complaints	.58/.75	.12/.97	.71** (.59–.83)	3	3
Suicide ideation/suicide attempts (K-SADS-PL)					
MAYSI-2 suicide ideation	.67/.79	.43/.91	.79*** (.72–.85)	3	2
French/Italian-speaking subsample					
Any substance use (K-SADS-PL)					
MAYSI-2 alcohol/drug use	.79/.71	.30/.96	.86*** (.77–.94)	3	4
Any disruptive behavior (K-SADS-PL)					
MAYSI-2 angry-irritable	.57/.56	.24/.84	.59 ns (.45–.73)	(6)	5
Any affective (K-SADS-PL)					
MAYSI-2 depressed-anxious	.56/.60	.33/.80	.56 ns (.44–.68)	(5)	3
MAYSI-2 somatic complaints	.52/.37	.22/.69	.49 ns (.36–.62)	(3)	3
Any anxiety (K-SADS-PL)					
MAYSI-2 depressed-anxious	.38/.54	.24/.69	.50 ns (.37–.62)	(5)	3
MAYSI-2 somatic complaints	.59/.39	.27/.71	.48 ns (.36–.60)	(3)	3
Suicide ideation/suicide attempts (K-SADS-PL)					
MAYSI-2 suicide ideation	.63/.69	.48/.81	.68** (.56–.80)	2	2

*MAYSI-2* Massachusetts Youth Screening Instrument-second version, *K-SADS-PL* Schedule for Affective Disorders and Schizophrenia for School-Age Children Present and Lifetime version, *Sens* sensitivity rate, *Spec* specificity rate, *PPV* positive predictive value, *NPV* negative predictive value, *AUC* area under the curve, *CI* confidence interval

*p < .05; ** *p* < .01; *** *p* < .001; *ns* not significant

^a^The optimal ‘caution’ cut-off scores are based on the difference between the sensitivity and specificity value to find the cut-off score with the lowest difference between both values

^b^The current cut-off scores are based on the results of the USA National Norms Study for the MAYSI-2 [17]

ROC analyses on the French/Italian-speaking subsample (Table 2) showed that any substance use disorder was accurately identified by the alcohol/drug use scale, and that the suicide ideation scale accurately identified suicide ideation/suicide attempts. The ROC analyses on the French/Italian speaking subsample revealed that the MAYSI-2 scales did not accurately identify any affective disorder, any disruptive disorder or any anxiety disorder. In the French/Italian-speaking subsample, compared to the current ‘caution’ cut-off scores, a deviant optimal ‘caution’ cut-off score was found for the alcohol/drug use scale (optimal ‘caution’ cut-off score with regard to any substance use: 3; current ‘caution’ cut-off score: 4).

ROC analyses on the boys’ and girls’ (Table 3) subsample showed that for boys and girls any substance use disorder was accurately identified by the alcohol/drug use scale. The angry-irritable scale accurately identified any disruptive disorder for boys and girls. The depressed-anxious scale accurately identified any affective disorder for boys, whereas no MAYSI-2 scale accurately identified any affective disorder for girls. For boys, any anxiety disorder was accurately identified by the depressed-anxious and somatic complaints scales; for girls, no MAYSI-2 scale yielded a significant AUC value higher than 0.50 to identify any anxiety disorder. For both boys and girls; suicide ideation/suicide attempts were accurately identified by the suicide ideation scale. In the boys’ subsample, compared to the current ‘caution’ cut-off scores, a deviant optimal ‘caution’ cut-off score was found for the somatic complaints scale (optimal ‘caution’ cut-off score with regard to any anxiety: 2; current ‘caution’ cut-off score: 3). In the girls’ subsample, compared to the current ‘caution’ cut-off scores, deviant optimal ‘caution’ cut-off scores were found for the angry-irritable scale (optimal ‘caution’ cut-off score with regard to any disruptive: 6; current ‘caution’ cut-off score: 5) and the suicide ideation scale (optimal ‘caution’ cut-off score with regard to suicide ideation/suicide attempts: 3; current ‘caution’ cut-off score: 2).

**Table 3:** Receiver operating characteristics estimates and optimal ‘caution’ cut-off scores of MAYSI-2 scales (Boys and Girls)

	Sens/Spec	PPV/NPV	AUC (95% CI)	Optimal cut-off^a^	Current cut-off^b^
Boys					
Any substance use (K-SADS-PL)					
MAYSI-2 alcohol/drug use	.78/.75	.38/.94	.84*** (.79–.88)	4	4
Any disruptive behavior (K-SADS-PL)					
MAYSI-2 angry-irritable	.51/.55	.55/.51	.58* (.51–.64)	5	5
Any affective (K-SADS-PL)					
MAYSI-2 depressed-anxious	.59/.61	.19/.91	.60* (.50–.70)	3	3
MAYSI-2 somatic complaints	.46/.57	.14/.88	.51 ns (.41–.60)	(2)	3
Any anxiety (K-SADS-PL)					
MAYSI-2 depressed-anxious	.71/.61	.14/.96	.69** (.59–.79)	3	3
MAYSI-2 somatic complaints	.75/.59	.14/.96	.68** (.57–.78)	2	3
Suicide ideation/suicide attempts (K-SADS-PL)					
MAYSI-2 suicide ideation	.69/.72	.33/.92	.73*** (.64–.82)	2	2
Girls					
Any substance use (K-SADS-PL)					
MAYSI-2 alcohol/drug use	.72/.74	.28/.95	.82*** (.74–.90)	4	4
Any disruptive behavior (K-SADS-PL)					
MAYSI-2 angry-irritable	.66/.67	.54/.77	.70*** (.62–.79)	6	5
Any affective (K-SADS-PL)					
MAYSI-2 depressed-anxious	.50/.61	.26/.82	.59 ns (.49–.69)	(5)	3
MAYSI-2 somatic complaints	.53/.44	.20/.77	.50 ns (.39–.62)	(3)	3
Any anxiety (K-SADS-PL)					
MAYSI-2 depressed-anxious	.54/.61	.21/.87	.59 ns (.46–.71)	(5)	3
MAYSI-2 somatic complaints	.67/.46	.19/.88	.60 ns (.48–.72)	(3)	3
Suicide ideation/suicide attempts (K-SADS-PL)					
MAYSI-2 suicide ideation	.65/.66	.48/.79	.74*** (.65–.82)	3	2

*MAYSI-2* Massachusetts Youth Screening Instrument-second version, *K-SADS-PL* Schedule for Affective Disorders and Schizophrenia for School-Age Children Present and Lifetime version, *Sens* sensitivity rate, *Spec* specificity rate, *PPV* positive predictive value, *NPV* negative predictive value, *AUC* area under the curve, *CI* confidence interval

*p < .05; ** *p* < .01; *** *p* < .001; *ns* not significant

^a^The optimal ‘caution’ cut-off scores are based on the difference between the sensitivity and specificity value to find the cut-off score with the lowest difference between both values

^b^The current cut-off scores are based on the results of the USA National Norms Study for the MAYSI-2 [17]

## Discussion

The current study estimated the diagnostic performance and optimal ‘caution’ cut-off scores of the MAYSI-2 in a sample of Swiss youths (i.e., French, German and Italian language regions) in welfare and juvenile justice institutions. As it has been reported [17, 26] that gender may influence the accuracy of self-report measures (e.g., girls tend to reveal their feelings on self-report scales more readily than boys), the other main purpose of the current study was to identify potential gender differences in the diagnostic performance and optimal ‘caution’ cut-off scores of the MAYSI-2 in a Swiss sample.

### Diagnostic performance

Within all of the subsamples, except in the French/Italian-speaking subsample, the MAYSI-2 scales alcohol/drug use and suicide ideation revealed significant AUC values above .70 indicating these scales are adequately precise [28]. These results indicate that the MAYSI-2 is able to identify youths who may be a danger to themselves and are in need of *direct* attention (i.e., youths with alcohol/drug problems and/or youths with suicidal ideation).

The results of the ROC analyses on the total sample, the German-speaking and the boy’s subsamples demonstrate that nearly all *homotypic* mappings of MAYSI-2 scales onto (cluster of) psychiatric disorders revealed above chance level accuracy (i.e., significant AUC values greater than .50).

Surprisingly, within the French/Italian-speaking and girl’s subsample, no significant AUC value higher than .50 was found for the MAYSI-2 scales depressed-anxious and somatic complaints. However, this finding should be interpreted with caution as low power (due to the relatively small number of youths from the French/Italian-speaking subsample (*n* = 105) and girls (*n* = 149) may have influenced the results. In addition, the MAYSI-2 somatic complaints scale did not reveal a significant AUC value for any affective disorder within any of the subsamples (i.e., total sample, German-speaking subsample, French/Italian-speaking subsample, boys and girls). The deviant diagnostic performance of this scale may be explained by the relatively small number of youths in some of the subsamples (i.e., French/Italian-speaking subsample and girls), as above mentioned. In addition, although the MAYSI-2 scale somatic complaints asks about bodily aches and pains that tend to occur with anxiety or affective disorders, it is possible that the MAYSI-2 scale somatic complaints does not discriminate well between bodily aches and pains that tend to occur with anxiety or affective disorders and bodily aches and pains related to a physical illness in a sample of Swiss youth in welfare and juvenile justice institutions.

### Optimal ‘caution’ cut-off scores

The optimal ‘caution’ cut-off scores derived from the ROC curve for predicting (cluster of) psychiatric disorders were, for several MAYSI-2 scales, comparable to the USA norm-based ‘caution’ cut-off scores (e.g., alcohol/drug use in all subsamples, angry-irritable in total sample, German-speaking and boys’ subsample). For some MAYSI-2 scales, however, higher optimal ‘caution’ cut-off scores were found. For example; compared to the USA norm-based ‘caution’ cut-off score, in the girls’ subsample a higher optimal ‘caution’ cut-off score was found for the MAYSI-2 scales angry-irritable and suicide ideation. The optimal ‘caution’ cut-off scores in the girls’ subsample for these MAYSI-2 scales were also higher than the optimal ‘caution’ cut-off scores for these scales in the boys’ subsample. This finding, which is consistent with earlier research [17, 26], raises questions about the response style of boys versus girls. It could be that Swiss girls have a higher threshold to report *clinically relevant* angry-irritable and suicidal behavior than American girls and boys. For example, the target population for which the MAYSI-2 was intended differs between the USA and Switzerland (e.g., due to a higher level of violence, harsh punishment, and more incarceration for youths [7, 25]). As a consequence, it may be that girls in the Swiss welfare and juvenile justice institutions have less severe mental health problems than girls in the USA juvenile justice system and therefore need to report more of their problems to reach the *clinically relevant* ‘caution’ cut-off score. In addition, it has been found that girls have higher rates of self-reported suicidal behavior than boys [29]; however, mortality from suicide is typically lower for girls than for boys [30]. In addition, as girls tend to reveal their feelings on self-report scales more readily than boys [17, 26], raising the ‘caution’ cut-off score for girls, compared to boys, on the abovementioned MAYSI-2 scales seems preferable in order to detect their *clinically relevant* potential emotional or behavioral problems adequately.

When determining the optimal ‘caution’ cut-off scores for the MAYSI-2 scale somatic complaints, it would be appropriate to select the optimal ‘caution’ cut-off scores based on the *homotypic* mappings that revealed above chance level accuracy (i.e., significant AUC values greater than 0.50). For example, in the total sample the optimal ‘caution’ cut-off score for somatic complaints with regard to any anxiety disorder would be appropriate. When determining the optimal ‘caution’ cut-off scores for the MAYSI-2 scale depressed-anxious, it would be appropriate to select the optimal ‘caution’ cut-off scores based on the lowest difference between the sensitivity and specificity value. For example, in the German-speaking subsample the optimal ‘caution’ cut-off score for depressed-anxious with regard to any affective disorder would be appropriate.

Furthermore, lowering the ‘caution’ cut-off score on the MAYSI-2 scale alcohol/drug use from 4 to 3 for the French/Italian subsample would increase the sensitivity rate (from .71 to .79). Consequently, more youths with the presence of any substance use disorder will be screened as such and less youths with the presence of any substance use disorder will be screened as not having any substance use disorder. While, on the other hand it would decrease the specificity rate (from .82 to .71). Meaning that less youths with the absence of any substance use disorder will be screened as such and more youths with the absence of any substance use disorder will be screened as having any substance use disorder. Thus, lowering the ‘caution’ cut-off score on the MAYSI-2 scale alcohol/drug use from 4 to 3 for the French/Italian subsample would imply that more youths will receive the correct special attention (i.e., provide additional psychological assessment, increased staff attention, close monitoring within the facility in order to prevent harm to the youth or others, or emergency mental health services) for their substance disorder, however more youths who are not in need of this special attention would also receive this attention.

In addition, raising the ‘caution’ cut-off score on the MAYSI-2 scale suicide ideation from 2 to 3 for the German subsample would decrease the sensitivity rate (from .80 to .67). Consequently, less youths with the presence of suicide ideation/suicide attempts will be screened as such and more youths with the presence of suicide ideation/suicide attempts will be screened as not having suicide ideation/suicide attempts. While, on the other hand it would increase the specificity rate (from .67 to .79). Meaning that more youths with the absence of suicide ideation/suicide attempts will be screened as such and less youths with the absence of suicide ideation/suicide attempts will be screened as having suicide ideation/suicide attempts. Thus, raising the ‘caution’ cut-off score on the MAYSI-2 scale suicide ideation from 2 to 3 for the German subsample would imply that less youths will receive the correct special attention (i.e., provide additional psychological assessment, increased staff attention, close monitoring within the facility in order to prevent harm to the youth or others, or emergency mental health services) for their suicide ideation/suicide attempts, however less youths who are not in need of this special attention would also receive this attention.

### Limitations

There are a few limitations of our study to mention. First, in the current study, the MAYSI-2 scales thought disturbance (a reliable scale only for boys) and traumatic experiences (no current ‘caution’ cut-off score determined [17]; were not included. Second, in the current study we did not test the diagnostic performance and optimal ‘caution’ cut-off scores of the MAYSI-2 across diverse ethnic subgroups. Because welfare and juvenile justice institutions in Switzerland are dealing with youths from ethnic subgroups that differ from those typically seen in the USA (e.g., Turkish versus African-American youths), future studies are critical to test whether the MAYSI-2 can be used within these subgroups. Third, we related the MAYSI-2 scale angry-irritable to any disruptive disorder. However, it should be emphasized that many youths may show angry or irritable behavior without being disruptively disordered. For example, irritable behavior may also be a symptom of a generalized anxiety disorder [31]. Fourth, in the original Massachusetts Study on which the USA National Norms Study for the MAYSI-2 was based [17], scales of the CBCL, YSR [Child Behavior Checklist, Youth Self-Report; 13, 14] and the Millon Adolescent Clinical Inventory (MACI) [32] were used to determine the diagnostic performance and the ‘caution’ cut-off scores, whereas in the current study (cluster of) psychiatric disorders were used. Relating the MAYSI-2 to (cluster of) psychiatric disorders may have been a strict way to estimate the diagnostic performance and the ‘caution’ cut-off scores of the instrument. Originally, the MAYSI-2 was not developed to diagnose specific psychiatric disorders; however its aim to screen for youths who may have severe psychiatric complaints indicates that MAYSI-2 scale scores are at least related to psychiatric disorders. Fifth, due to the study design and due to conflicting schedules of youths; the time that passed between facility intake and the MAYSI-2 screening, and the time that passed between the MAYSI-2 screening and the K-SADS-PL interview was different for all youths and could have influenced the results. Lastly, we should note that several findings of the current study should be interpreted with caution as low power may have influenced the results.

## Conclusions

With adjusted optimal ‘caution’ cut-off scores, the MAYSI-2 screens potential emotional or behavioral problems well in a sample of Swiss youths in welfare and juvenile justice institutions. As for choosing the optimal ‘caution’ cut-off score for the MAYSI-2 both language as well as gender seem to be of importance; the results of this study indicate the need to test the diagnostic performance and optimal ‘caution’ cut-off scores of the MAYSI-2 more elaborately in larger differentiated language samples in Europe. Furthermore, when choosing the optimal ‘caution’ cut-off score for the MAYSI-2, the consequences (e.g., detecting youths in need of special attention versus missing youths in need of this special attention) of raising or lowering a certain cut-off score should be kept in mind. In addition, given that caution should be taken into account when using the MAYSI-2 in non-juvenile justice subsamples [i.e., reason for stay because of *civil law measure* or because of *other reasons*; 18], future studies are critical to test the diagnostic performance and optimal cut-off scores of the MAYSI-2 within those non-juvenile justice subsamples. Our findings also emphasize the importance of using caution when setting subsample-specific ‘caution’ cut-off scores. For example, raising ‘caution’ cut-off scores on MAYSI-2 scales (e.g., suicide ideation for the German subsample or angry-irritable and suicide ideation for girls) will consequently influence the sensitivity and specificity rates of these scales; which will influence the number of youths with the presence or absence of a certain psychiatric disorder who will be screened as such. This in turn will influence the decisions which will be made, such as whether or not refer youths to available treatment services. In addition, a second consequence has to do with cross-national comparisons between studies. When setting subsample-specific ‘caution’ cut-off scores for the MAYSI-2, it will be more difficult to compare results across studies in other countries or cultures. However, subsample-specific ‘caution’ cut-off scores for the MAYSI-2 may improve screening youths entering welfare and juvenile justice institutions. It may contribute to signalizing the special needs (i.e., provide additional psychological assessment, increased staff attention, close monitoring within the facility in order to prevent harm to the youth or others, or emergency mental health services) of these youths more precisely and, on the long run, may contribute to offering them suitable treatment services intended to reduce their mental health problems [17]. Lastly, our findings also emphasize the importance of keeping in mind that when screening youths in welfare and juvenile justice institutions not only to focus on those who score above a certain cut-off score. When youths are screened in welfare and juvenile justice institutions their (self-reported) emotional or behavioral problems may fluctuate depending on a variation of internal and/or external factors (e.g., developmental level, motivation for treatment, or crisis caused by the situation). Consequently, it seems important, when determining the diagnostic needs of these youths, not to rely on the results of their self-reported screening results only but also to use alternative resources of information (e.g., clinical view of therapist, observations or repeated measures).

## Abbreviations

AUC: Area under the curve
CBCL: Child behavior checklist
CI: Confidence interval
ICD-10: International statistical classification of diseases and related health problems, 10^th^ revision
K-SADS-PL: Schedule for Affective Disorders an Schizophrenia for School-Age Children, Present and Lifetime version
MACI: Million adolescent clinical inventory
MAYSI-2: Massachusetts youth screening instrument - second version
NPV: Negative predictive value
PPV: Positive predictive value
PTSD: Posttraumatic stress disorder
ROC: Receiver-operating characteristic
Sens: Sensitivity rate
Spec: Specificity rate
SPSS: Statistical package for social sciences
USA: United States of America
YABCL: Young adult behavior checklist
YSR: Youth self-report
